# Electroconvulsive Therapy-Induced Changes in Functional Brain Network of Major Depressive Disorder Patients: A Longitudinal Resting-State Electroencephalography Study

**DOI:** 10.3389/fnhum.2022.852657

**Published:** 2022-05-18

**Authors:** Shuting Sun, Peng Yang, Huayu Chen, Xuexiao Shao, Shanling Ji, Xiaowei Li, Gongying Li, Bin Hu

**Affiliations:** ^1^Brain Health Engineering Laboratory, School of Medical Technology, Beijing Institute of Technology, Beijing, China; ^2^Shandong Daizhuang Hospital, Jining, China; ^3^Gansu Provincial Key Laboratory of Wearable Computing, School of Information Science and Engineering, Lanzhou University, Lanzhou, China; ^4^Shandong Academy of Intelligent Computing Technology, Jinan, China; ^5^Department of Psychiatry, Huai’an Third People’s Hospital, Huai’an, China; ^6^Chinese Academy of Sciences Center for Excellence in Brain Science and Intelligence Technology, Shanghai Institutes for Biological Sciences, Chinese Academy of Sciences, Shanghai, China; ^7^Joint Research Center for Cognitive Neurosensor Technology of Lanzhou University and Institute of Semiconductors, Chinese Academy of Sciences, Lanzhou, China; ^8^Open Source Software and Real-Time System, Lanzhou University, Ministry of Education, Lanzhou, China

**Keywords:** electroconvulsive therapy, electroencephalography, major depressive disorder, functional connectivity, graph theory analysis

## Abstract

**Objectives:**

Several studies have shown abnormal network topology in patients with major depressive disorder (MDD). However, changes in functional brain networks associated with electroconvulsive therapy (ECT) remission based on electroencephalography (EEG) signals have yet to be investigated.

**Methods:**

Nineteen-channel resting-state eyes-closed EEG signals were collected from 24 MDD patients pre- and post-ECT treatment. Functional brain networks were constructed by using various coupling methods and binarization techniques. Changes in functional connectivity and network metrics after ECT treatment and relationships between network metrics and clinical symptoms were explored.

**Results:**

ECT significantly increased global efficiency, edge betweenness centrality, local efficiency, and mean degree of alpha band after ECT treatment, and an increase in these network metrics had significant correlations with decreased depressive symptoms in repeated measures correlation. In addition, ECT regulated the distribution of hubs in frontal and occipital lobes.

**Conclusion:**

ECT modulated the brain’s global and local information-processing patterns. In addition, an ECT-induced increase in network metrics was associated with clinical remission.

**Significance:**

These findings might present the evidence for us to understand how ECT regulated the topology organization in functional brain networks of clinically remitted depressive patients.

## Introduction

Major depressive disorder (MDD) is a complex psychiatry disorder that includes three categories of clinical manifestations: disorders of mood, cognitive function, and neurovegetative functions (i.e., sleep and appetite function) (Lisanby, 2007), which severely affects a patient’s quality of life and can even lead to suicide. The prevalence of depression is about 2–4% worldwide and 1.7–2% in China alone (Gu et al., 2013). Hence, effective treatment of depression is a major public health challenge (Sartorius, 2001). Nowadays, compared to some commonly available treatments, such as cognitive behavioral therapy (Siegle et al., 2006; Ritchey et al., 2011) and antidepressant medication (Spronk et al., 2011), electroconvulsive therapy (ECT) (Bouckaert et al., 2016; Yrondi et al., 2018; Takamiya et al., 2019) has long been considered to be the most effective and rapid treatment for depression, and around 70–90% of the depressed patients showed an improvement when treated with ECT (Kellner et al., 2014). However, the mechanisms underlying the action of ECT are still not known.

Mounting evidence suggests that MDD is a system-level disorder that is associated with the dysfunction of neuronal network activity across multiple brain regions (Mayberg et al., 2005). Some structural and functional neuroimaging studies have investigated ECT-induced changes in the structural or functional connectivity of depression by conducting a longitudinal study (Abbott et al., 2013; Bouckaert et al., 2016; Wei et al., 2018). Functional connectivity refers to the degree of temporal correlation between neural signals coming from two different regions (Fingelkurts et al., 2005). Electroencephalogram (EEG), owing to its advantages such as high temporal resolution, non-invasive nature, and a relatively inexpensive technique, becomes a preferable choice for computing functional connectivity, which can be widely applied in clinical and research fields (Sakkalis, 2011; van Diessen et al., 2015).

Existing studies indicated that ECT regulated resting-state EEG oscillatory patterns in the frontal lobe (Farzan et al., 2014) or central nodes of the default mode network (DMN) (Takamiya et al., 2019). Some studies have found that ECT decreased EEG complexity in the responded patients based on the multi-scale entropy method (Okazaki et al., 2013; Farzan et al., 2017). In addition, other EEG studies using low-resolution electromagnetic tomography (LORETA) analysis revealed that ECT-induced theta changes in the subgenual anterior cingulate cortex (ACC) (Reischies et al., 2005) were correlated with decreased depressive scale scores (McCormick et al., 2009). However, compared to neuroimaging studies, the number of studies examining the alteration in ECT-induced functional connectivity networks based on EEG is still small. Only a few EEG studies based on functional connectivity methods reported that ECT changed phase synchronization in the beta/delta frequency band (Deng et al., 2015; Takamiya et al., 2019). Currently, graph theory analysis based on functional connectivity is a simple and straightforward approach to evaluate the topological structure of complex brain networks (He and Evans, 2010; Rubinov and Sporns, 2010). Moreover, graph measures have also been applied to reveal the changes in network topology following seizure therapy. One EEG study that used the graph theory technique reported a decrease of functional connectivity in the beta frequency band and deviation from the small-world architecture, including increased characteristic path length and decreased clustering coefficient, in depressive patients who received a single session of treatment for seizure therapy [either ECT or magnetic seizure therapy (MST)] (Deng et al., 2015). Similarly, another study of EEG based on graph theory analysis suggested an increased clustering coefficient and decreased characteristic path length in the theta frequency band and decreased clustering coefficient in the beta frequency band in depressive patients who received a series of MST (Hill et al., 2021). These results indicate that seizure therapy has the potential to modulate the functional topology of the brain, but larger studies are needed to evaluate the effects of single therapy alone to confirm these preliminary findings. Hence, to expand current research, this study will analyze the change mode of the whole brain topology of depressive patients pre- and post-ECT treatment from the perspective of a functional brain network based on EEG signals.

In this prospective, longitudinal study, we used resting-state EEG signals to examine the functional brain networks in patients with MDD (*N* = 24) at two time points: (1) prior to ECT and (2) within 7–10 days of completion of ECT series. The first objective of this study was to explore the differences between brain network topology structure pre- and post-ECT in patients with MDD, particularly based on the following three aspects: changes in functional connectivity, changes in graph theoretical measures (e.g., local/global efficiency), and changes in hubs distribution. The second objective of this study was to investigate whether the changes in these network measures correlated with clinical response.

## Materials and Methods

### Participants

Twenty-four patients (men/women: 15/9, mean age: 33.54 ± 13.75 years) with MDD were recruited from the Jining Daizhuang Hospital, Shandong, China. The study was approved by the Local Research Ethics Committee, and written informed consent was obtained from all the subjects before the ECT treatment began. The inclusion criteria were as follows: (1) all MDD patients met the diagnostic criteria for the Chinese Guideline for Prevention and Treatment of Depression and met the clinical indications of modified ECT therapy, and the score of the 17-Hamilton Depression Rating Scale (HAMD-17) (Hamilton, 1967) was ≥24; (2) the age should be between 18 and 65 years with a primary or higher educational level; and (3) the MDD patients voluntarily agreed to receive the modified ECT therapy and signed the informed consent. If the condition seriously affected the capacity for civil conduct, the guardian should decide whether to accept the modified ECT therapy and sign informed consent. The exclusion criteria for all the MDD patients were as follows: (1) suffering from other mental disorders (e.g., delusional disorder and post-traumatic stress disorder); (2) having physical and nervous system diseases (e.g., cardiovascular and cerebrovascular diseases or Parkinson’s disease); (3) struggling with any psychoactive substance abuse or dependence (except nicotine); (4) diagnosed with epilepsy, brain tumors, and brain trauma caused by the disorders of consciousness, with loss of consciousness for more than 5 min, and history of other neurological diseases; (5) the patients believed to have somatic diseases that led to abnormal EEG results, according to the assessment of EEG specialists; and (6) having received ECT treatment within the last 3 months. Depressive severity was assessed in patients using the HAMD-17 Scale within 3 days prior to the first ECT session and 7–10 days after the last ECT session.

### Electroconvulsive Therapy Procedure

Electroconvulsive therapy was administered using a THYMATRON System IV device (Somatics, LLC, Lake Bluff, IL, United States). All the 24 MDD patients received bitemporal ECT stimulus delivery with a constant-current brief pulse (1 ms). The duration of the electrical stimulation depended on the age of the patients, which was typically 5 s and sometimes 7–8 s for elderly patients. Treatments were performed 3–4 times a week and were continued until a plateau was reached, and no further improvement occurred. Atropine (0.5 mg) was used to reduce respiratory secretions, propofol (1.5–2 mg/kg) was used for general anesthesia, and succinylcholine (1 mg/kg) was used to induce muscle relaxation. In this experiment, blood pressure, heart rate, and oxygen saturation levels were continuously monitored.

### Electroencephalography Acquisition and Pre-processing

The first EEG recording was performed within 1–3 days of the first ECT session, and the second EEG recording was done within 7–10 days after the completion of the last ECT session. EEG data were collected on Nihon Kohden EEG machines (Neurofax EEG-1200C) by trained technicians. Ten minutes of eyes-closed resting-state EEG data were recorded from 19 scalp locations according to the standard international 10/20 system (Fp1/2, F3/4, C3/4, P3/4, O1/2, F7/8, T3/4, T5/6, Fz, Cz, and Pz) referenced to linked ears. The sampling frequency was 500 Hz. Electrode impedance was kept below 5 kΩ.

The Automagic toolbox (Pedroni et al., 2019) was used for data import and pre-processing. To provide noise reduction, high-pass and low-pass filters were set with 1 Hz and 40 Hz cutoff frequencies, respectively. The Fz channel signal was used for electrooculogram (EOG) regression (Croft and Barry, 2000) to eliminate the EOG of other channels. Then Multiple Artifact Rejection Algorithm (MARA) (Winkler et al., 2011) was adopted to reject other artifacts. The reconstructed and cleaned EEG signals were re-reference to the average reference. Next, the EEG recordings were continuously divided into 2 s for each epoch. For all the epochs, we calculated three indicators: ratio of data with overall high amplitude (OHA), ratio of time points of high variance (THV), and ratio of channels of high variance (CHV). Then, these epochs were classified into “Good,” “Ok,” or “Bad” according to the indicators and default threshold. If OHA < 0.1, THV < 0.1, and CHV < 0.15, the data were classified as “Good;” if 0.1 < OHA < 0.2, 0.1 < THV < 0.2, and 0.15 < CHV < 0.3, the data were classified as “Ok”; and if 0.2 < OHA < 30, 0.2 < THV < 15, and 0.3 < CHV < 15, the data were classified as “Bad.” Finally, for each subject, the first 60 epochs marked as “Good” were selected for the subsequent analysis. As a note, we chose 16 electrodes (Fp1/2, F3/4, C3/4, P3/4, O1/2, F7/8, T3/4, and T5/6) to construct the functional brain network, which has been used previously in the studies on depression (Akdemir Akar et al., 2015; Li X. et al., 2016). The tool used for data processing was MATLAB R2017a.

### Functional Connectivity Matrices

The first step in constructing a functional brain network is to calculate the connectivity matrix, in which each node represents the electrode and each edge represents the connection strength between the electrodes. To construct a functional connectivity matrix, coherence (Coh), imaginary part of coherence (ICoh), and phase lag index (PLI) were used in this study (a brief description of the three coupling methods is given in Supplementary Section 1). We calculated the Coh/ICoh/PLI matrix for 60 epochs, and then all the Coh/ICoh/PLI matrices were averaged across all epochs to obtain the functional connectivity matrix for each subject. The dimension of the functional connectivity matrix is 16 * 16, where 16 is the number of electrode channels. The frequency bands of analysis include delta band (1–4 Hz), theta band (4–8 Hz), alpha band (8–13 Hz), and beta band (13–30 Hz).

### Binarization Methods

The second step in constructing a functional brain network is to extract a binary network from the weighted connectivity matrices obtained by the above-mentioned methods. Original weighted connectivity matrices usually present some weak and spurious connections, so we should use a method to remove the non-significant links and alleviate the noise level. In this study, we adopted state-of-the-art non-arbitrary unbiased binarization approaches Cluster-Span Threshold (CST) and Minimum Spanning Tree (MST), which have been proved to show good performance in the modeling of EEG network topology (Smith et al., 2015, 2017; Li X. et al., 2017) (a brief description of the two binarization methods is given in Supplementary Section 2).

### Network Metrics

In the following experiments, we used the graph theory analysis method to quantitatively evaluate the topological properties of these binary brain networks, particularly from information integration and segregation. In this study, we computed global efficiency (GE) (Latora and Marchiori, 2001), edge betweenness centrality (EBC) (Freeman, 1978), node betweenness centrality (NBC) (Freeman, 1978), local efficiency (LE) (Latora and Marchiori, 2001), and mean degree (meanD) (Rubinov and Sporns, 2010) (mathematical formula for the network metrics is given in Supplementary Section 3). The network metrics were calculated by using Brain Connectivity Toolbox.^1^

In addition, hubs play a vital role in efficient communication and network resilience to insult (Bullmore and Sporns, 2009). Recent studies have shown abnormalities in the hub configuration (e.g., number and distribution of hubs) of brain functional networks in neuropsychiatric diseases (e.g., depression and schizophrenia) (Alexander-Bloch et al., 2013; Sun et al., 2019), which suggest an important association between hub regions and pathophysiological mechanisms. So this study also analyzed the characteristics of topology distribution of hubs in pre- and post-ECT patients with MDD. We computed hubs for the binary connectivity matrix of each subject; if the frequency of a node as the hub was greater than 30% of the number of subjects, the node was defined as the hub of the group. A node can be considered as a hub if its degree is at least one standard deviation above the mean degree of the network (Hosseini et al., 2012). The degree of a node is the number of links connected to it.

### Statistical Analysis

The functional connectivity values and network metrics were compared in pre- and post-ECT patients with MDD. The statistical threshold was calculated by performing a pseudo-paired *t*-test based on the permutation test (*n* = 10,000, *p* = 0.05). First, a *t*-value was calculated for each studied metric (functional connectivity values, GE, EBC, NBC, LE, and meanD) as an observed test statistic between pre- and post-ECT MDD groups. Next, each subject in the pre- and post-ECT MDD group was randomly shuffled, so after permutation, the number ratio of pre- and post-ECT MDD groups was still 24:24. The *t*-values were recalculated for the permutated groups 10,000 times, and the null distribution of test statistics was obtained for the group difference. Finally, the proportion of sampled permutations, where the *t*-values were greater than the observed test statistic, was determined as the *p*-value of the observed group difference. A level of significance was *p* < 0.05.

For the functional connectivity value or network metrics having significant differences, we conducted repeated measures correlation (rmcorr) (Bakdash and Marusich, 2017) based on bootstrapping (nrep = 1,000) to test if the change in these metrics was correlated to the change in the HAMD scores. Repeated measures correlation is suitable for analyzing the common linear association between paired repeated measures for multiple individuals, which has been adopted in the related studies (Nuninga et al., 2020; Gbyl et al., 2021; Takamiya et al., 2021). As an additional analysis, we also conducted a Pearson correlation analysis based on a permutation test (*n* = 10,000, *p* = 0.05) between changed values of measures (post-functional connectivity value/network metric subtract pre-functional connectivity value/network metric) and changed HAMD scores (pre-HAMD score subtract post-HAMD score). The method of Pearson correlation analysis, based on a permutation test, was similar to that of the pseudo-paired *t*-test based on a permutation test. The observed test statistic was defined as the correlation coefficient between the changed measures and changed HAMD scores. Like the Pearson correlation, the rmcorr coefficient (r_*rm*_) represents the linear correlation between two variables, and the value ranges from –1 to 1.

## Results

### Demographic Variables and Clinical Characteristics

**FIGURE 1:**
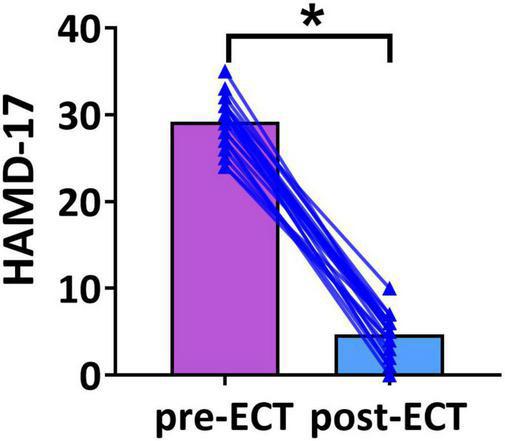
The Hamilton Depression Rating Scale-17 (HAMD-17) scores of pre- and post-ECT patients with MDD. The bar chart represents the mean values of HAMD-17 scores in pre- and post-ECT treatments. The * indicates a significant difference (*p* < 0.05).

Demographic variables and clinical characteristics of patients with MDD are presented in Table 1. The HAMD scores of pre- and post-ECT are shown in Figure 1. The HAMD scores of patients with MDD pre- and post-ECT were 29.21 ± 2.78 and 4.67 ± 2.35, respectively. According to previous treatment studies of depression, the response was defined as a reduction in the HAMD score of more than 50%, while remission was defined as the HAMD score less or equal to 7 (Keller, 2003; Brunoni et al., 2016; Wei et al., 2020). So in this study, the clinical response rate was 100% (24/24), and the clinical remission rate was 95.83% (23/24). The number of ECT treatments was 11.05 ± 1.56.

**TABLE 1:** Demographic variables and clinical characteristics of patients with MDD.

Characteristic	Remitted group
Age, years	33.54 ± 13.75
Education, years	9.43 ± 4.55
Gender (male/female)	15/9
Marital status (married/unmarried)	14/10
Age at onset, years	29.08 ± 13.62
Number of depressive episodes	2.42 ± 1.14
Duration of current episode, months	1.91 ± 2.36
Number of ECT treatments	11.05 ± 1.56
Time between the pre-EEG and the first ECT, days	1–3
Time between the last ECT and post-EEG, days	7–10
**HAMD-17 total score**	
Pre-ECT	29.21 ± 2.78
Post-ECT	4.67 ± 2.35

*MDD, major depressive disorder; HAMD-17, 17-Hamilton Depression Rating Scale, the values in the table are expressed as mean ± standard deviation.*

### Electroconvulsive Therapy-Induced Changes in Functional Connectivity

**FIGURE 2:**
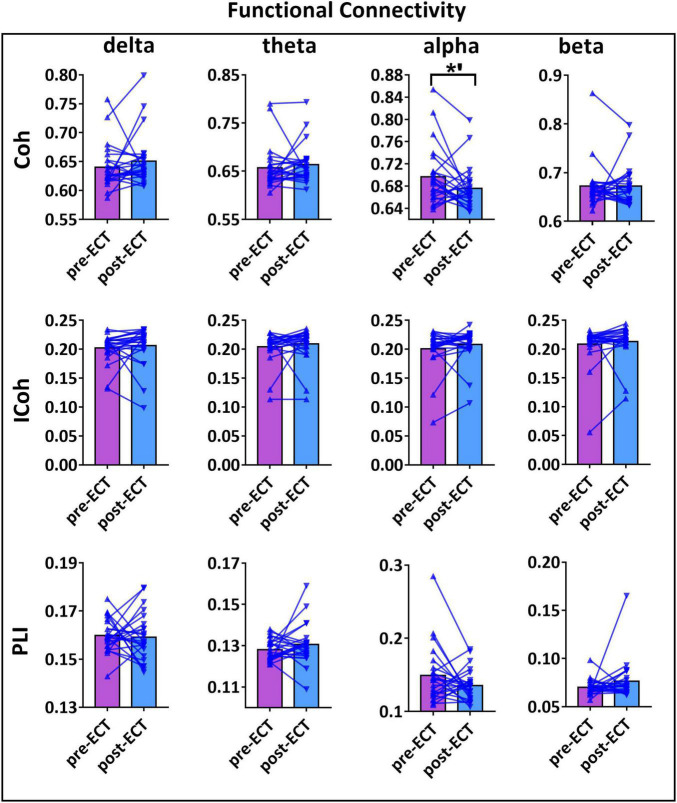
The functional connectivity (FC) values of pre- and post-ECT patients with MDD obtained by three coupling methods [coherence (Coh), imaginary part of coherence (ICoh), and phase lag index (PLI)] in delta, theta, alpha, and beta frequency bands. The bar chart represents the mean values of FC in pre- and post-ECT treatments. The *’ represent marginally significant difference (0.05 < *p* < 0.1, pseudo-paired *t*-test based on permutation test, *n* = 10,000, *p* = 0.05).

Longitudinal changes in the functional connectivity of patients with MDD pre- and post-ECT in delta, theta, alpha, and beta frequency bands are shown in Figure 2. There was a marginally reduced (*t*_23_ = 1.966, *p* = 0.062) functional connectivity post-ECT (0.676 ± 0.039) when compared to that pre-ECT (0.696 ± 0.054) in alpha frequency band, only when functional connectivity was calculated by Coh. No significant differences were found in other frequency bands based on the other two coupling methods (ICoh, PLI).

### Electroconvulsive Therapy-Induced Changes in Network Metrics

**FIGURE 3:**
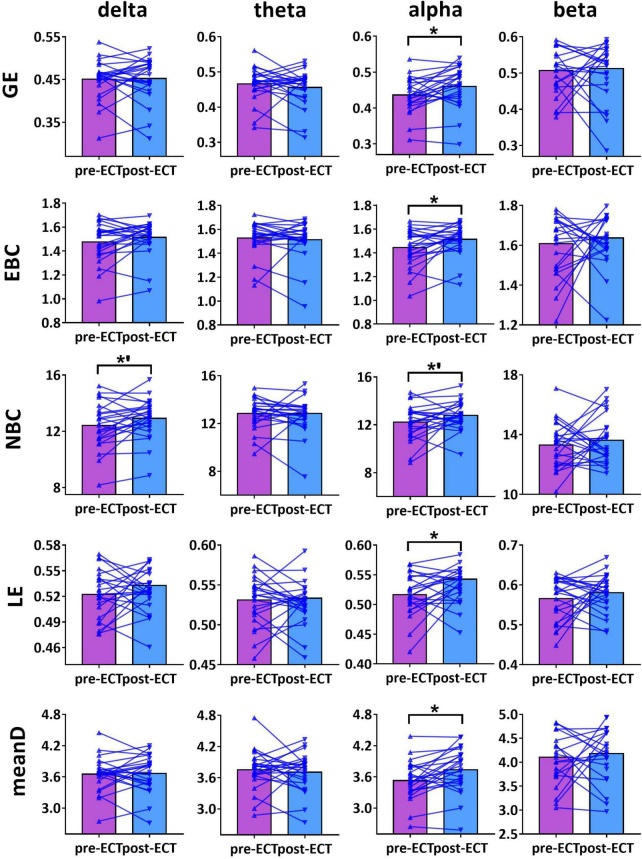
Network metrics of pre- and post-ECT patients with MDD obtained by coupling method Coh and binarization approach Cluster-Span Threshold (CST) in delta, theta, alpha, and beta frequency bands. Network metrics include global efficiency (GE), edge betweenness centrality (EBC), node betweenness centrality (NBC), local efficiency (LE), and mean degree (meanD). The bar chart represents the mean value of network metrics in pre- and post-ECT MDD patients. The * indicates a significant difference (*p* < 0.05), the *’ represent marginally significant difference (0.05 < *p* < 0.1), Pseudo-paired *t*-test was conducted based on permutation test, *n* = 10,000, *p* = 0.05.

**FIGURE 4:**
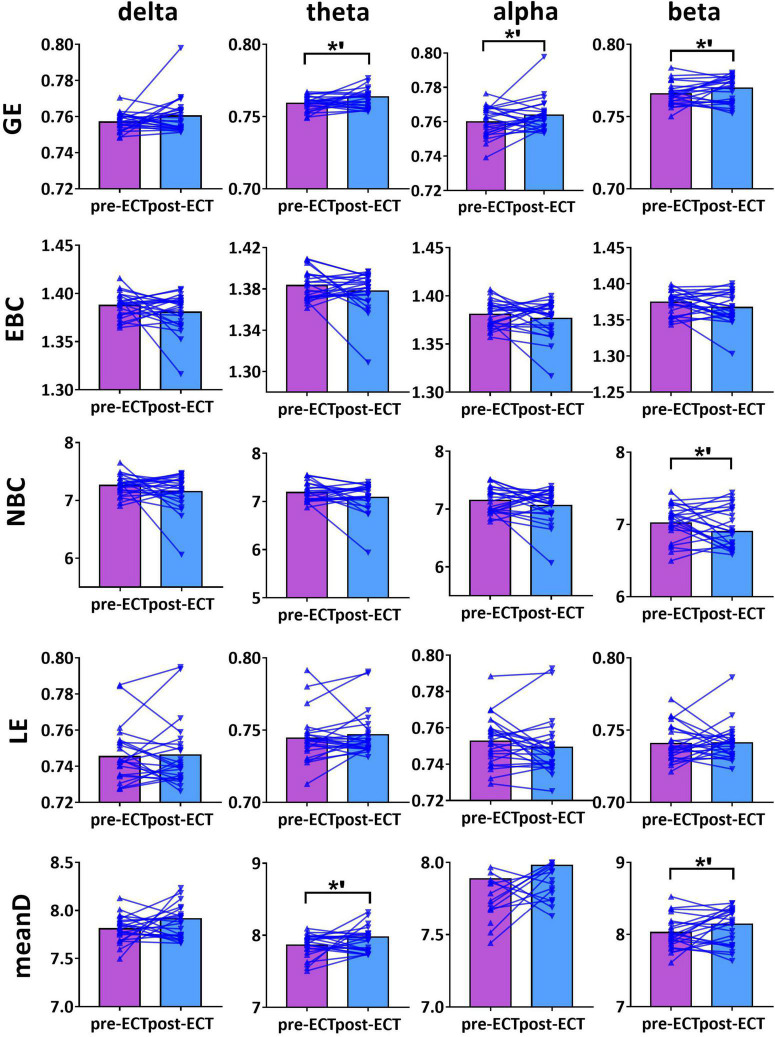
Network metrics of pre- and post-ECT patients with MDD obtained by coupling method ICoh and binarization approach CST in delta, theta, alpha, and beta frequency bands. Other descriptions are as in Figure 3.

**FIGURE 5:**
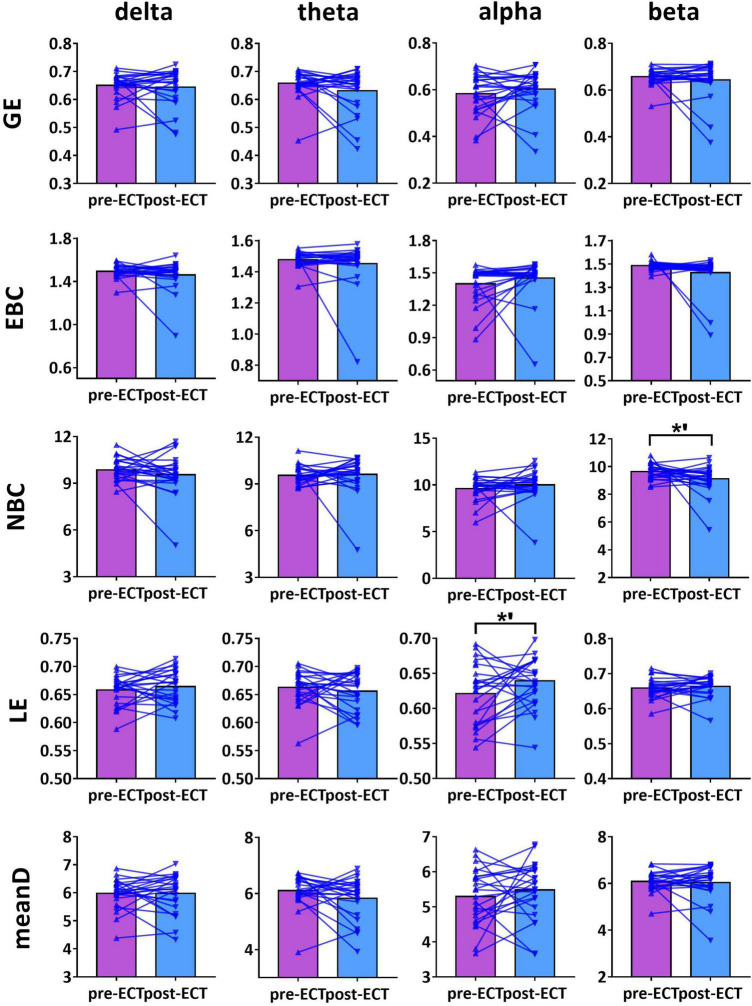
Network metrics of pre- and post-ECT patients with MDD obtained by coupling method PLI and binarization approach CST in delta, theta, alpha, and beta frequency bands. Other descriptions are as in Figure 3.

When CST was used for binarization, ECT-induced network metrics showed the following changes (Figures 3–5): for delta frequency band, when the connectivity values were estimated by Coh, there was a marginal increase in NBC (pre-ECT: 12.41 ± 1.64, post-ECT: 12.92 ± 1.40, *t*_23_ = 2.008, *p* = 0.057) (Figure 3).

For theta frequency band, when coupling method ICoh was used, there were marginal increases in GE (pre-ECT: 0.759 ± 0.005, post-ECT: 0.764 ± 0.011, *t*_23_ = 1.948, *p* = 0.064) and meanD (pre-ECT: 7.864 ± 0.161, post-ECT: 7.974 ± 0.288, *t*_23_ = 1.728, *p* = 0.097) (Figure 4).

For alpha frequency band, when coupling method Coh was used, network metrics GE (pre-ECT: 0.436 ± 0.050, post-ECT: 0.460 ± 0.057, *t*_23_ = 2.546, *p* = 0.018), EBC (pre-ECT: 1.443 ± 0.158, post-ECT: 1.513 ± 0.129, *t*_23_ = 2.467, *p* = 0.022), LE (pre-ECT: 0.517 ± 0.035, post-ECT: 0.543 ± 0.048, *t*_23_ = 2.425, *p* = 0.024), and meanD (pre-ECT: 3.529 ± 0.376, post-ECT: 3.734 ± 0.428, *t*_23_ = 2.934, *p* = 0.008) showed a significant increase after ECT treatment, and there was a marginal increase in NBC (pre-ECT: 12.224 ± 1.474, post-ECT: 12.785 ± 1.176, *t*_23_ = 1.821, *p* = 0.082) (Figure 3). Likewise, when the connectivity values were estimated by ICoh, there was a marginal increase in GE (pre-ECT: 0.760 ± 0.009, post-ECT: 0.764 ± 0.010, *t*_23_ = 1.780, *p* = 0.088) (Figure 4). When coupling method PLI was used, post-ECT patients with MDD exhibited marginal increase in LE (pre-ECT: 0.621 ± 0.043, post-ECT: 0.639 ± 0.037, *t*_23_ = 1.854, *p* = 0.077) (Figure 5).

For beta frequency band, when coupling method ICoh was used, there was a marginal increase in GE (pre-ECT: 0.766 ± 0.008, post-ECT: 0.770 ± 0.012, *t*_23_ = 1.820, *p* = 0.082) and meanD (pre-ECT: 8.026 ± 0.229, post-ECT: 8.141 ± 0.328, *t*_23_ = 1.861, *p* = 0.076). Network metric of NBC (pre-ECT: 7.020 ± 0.248, post-ECT: 6.902 ± 0.354, *t*_23_ = 1.716, *p* = 0.100) showed marginal reduction after ECT treatment (Figure 4). When PLI was used, a marginal reduction in NBC was also noticed (pre-ECT: 9.649 ± 0.561, post-ECT: 9.114 ± 1.008, *t*_23_ = 1.962, *p* = 0.062) (Figure 5).

However, we found that network metrics showed no significant differences in pre- and post-ECT patients with MDD in four bands when MST combined with Coh, ICoh, or PLI was used. In addition, the network sparsity of CST was significantly higher than that of MST (detailed results are provided in Supplementary Section 4).

### Electroconvulsive Therapy-Induced Alteration in Hubs Characteristic

**FIGURE 6:**
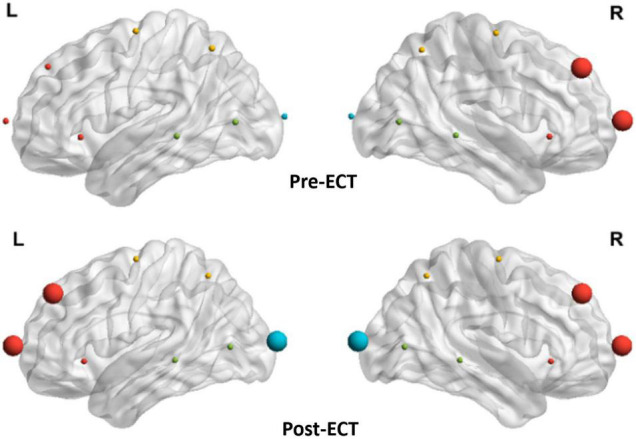
Distribution of hubs of pre- and post-ECT patients with MDD in the alpha band. The enlarged spheres represent the network hubs. Red color nodes represent the frontal lobe; yellow color nodes represent the central lobe; green color nodes represent the temporal lobe; and blue color nodes represent the parietal-occipital lobe.

As the network metrics of the alpha frequency band show a significant difference between pre- and post-ECT treatments, in the subsequent analysis, we investigated how ECT modulated the hubs distribution in the alpha frequency band. From Figure 6, the hubs in the pre-ECT patients with MDD were mainly distributed in the right frontal lobes (Fp2 and F4). However, the hubs in the post-ECT patients with MDD were mainly distributed in the frontal (Fp1/2 and F3/4) and occipital (O1/2) lobes.

### Correlation Between Changes in Network Metrics and Clinical Response

**FIGURE 7:**
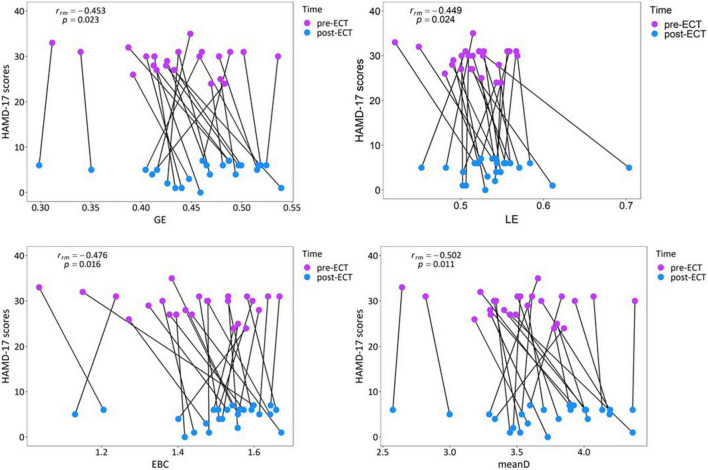
Relationship between the network metrics and the clinical response in individual patients with MDD in the alpha band.

According to the obtained statistical results of network metrics, correlation analysis was conducted between network metrics having significant differences (GE, LE, EBC, and meanD of alpha frequency band) and clinical response. Repeated measures correlation revealed that the increase in the values of network metrics GE [r_*rm*_(23) = −0.453, *p* = 0.023], LE [r_*rm*_(23) = −0.449, *p* = 0.024], EBC [r_*rm*_(23) = −0.476, *p* = 0.016], and meanD [r_*rm*_(23) = −0.502, *p* = 0.011] was moderately correlated to the decrease in the HAMD-17 scores after ECT treatment (Figure 7). Pearson correlation suggested that there was no significant correlation between these changed network metrics and changed HAMD-17 scores (Supplementary Section 5).

## Discussion

This study used functional connectivity and graph theory analysis to explore the topological changes in resting-state EEG functional brain network of depressive patients pre- and post-ECT treatment. For clinically remitted patients with a HAMD-17 score ≤ 7 and no relapse within 3 months, our main findings were as follows: (1) ECT marginally significantly decreased functional connectivity in the alpha frequency band. (2) ECT increased GE, EBC, LE, and meanD values, which revealed that depressive patients tended to have a superior network structure after ECT treatment. Also, changes in these network metrics had significantly moderate correlations with clinically remitted depressive symptoms in repeated measures correlation. (3) ECT changed the distribution of hubs, mainly in frontal and occipital lobes. Collectively, these findings provided insight into the role of the functional brain network in the underlying mechanism of action of ECT for the treatment of depressive patients.

In this pilot study, when Coh was used to calculate the functional connectivity value, there was a marginal but significant decrease in functional connectivity in the alpha frequency band after ECT treatment. Although normal subjects were not included in this study, the obtained results could be verified with the previous literature. Leuchter et al. (2012) used quantitative EEG coherence to examine differences in the resting-state functional connectivity between patients with MDD and healthy controls. Significantly higher overall coherence in the delta (0.5–4 Hz), theta (4–8 Hz), alpha (8–12 Hz), and beta (12–20 Hz) frequency bands were found in the subjects with MDD. Despite the contrary findings, most studies on depressive patients over the age of 30 years have reported increased functional connectivity (Bohr et al., 2012; Li et al., 2015). The increased functional connectivity of MDD patients indicated that the depressive patients had abnormally activated or overloaded brain activity (Rotenberg, 2004), which might result from the low efficiency of network organization. Moreover, the most significant increases in coherence were found in the alpha frequency band, which could be explained by the failure of the top-down control imposed by rhythmic alpha activity (Leuchter et al., 2012). In this study, post-ECT depressive patients had marginally significantly reduced functional connectivity in the alpha frequency band compared to the pre-ECT depressive patients. Hence, we could speculate that ECT treatment could modulate the abnormal brain pattern in depressive patients and improve the efficiency of brain information communication. Furthermore, a resting-state functional magnetic resonance imaging (fMRI) ECT study had proposed the “hyper-connectivity” hypothesis, which suggested that treatment responses during depressive episodes might be related to decreased seed-voxel connectivity within some brain networks (e.g., cognitive control network and DMN) (Sheline et al., 2010; Perrin et al., 2012). However, another fMRI ECT study showed that the functional connectivity of the left angular gyrus significantly increased after ECT in depression patients, which is accompanied by improved mood (Wei et al., 2018). A related EEG ECT study found increased functional connectivity in the theta frequency band between the posterior cingulate cortex and the anterior prefrontal cortex, and decreased functional connectivity in the beta frequency band between the posterior cingulate cortex and the temporal regions (Takamiya et al., 2019). So, from the inconsistent research results, we consider that the ECT treatment-induced increased or decreased functional connectivity might depend on the brain regions. Future studies should focus on the effect of ECT on functional connectivity of the whole and local brain regions to validate our findings and those of previous studies.

To objectively represent the topological structure of complex brain networks, we adopted two novel binarization methods, CST and MST, to construct the binary brain networks based on the functional connectivity matrix calculated by Coh, ICoh, and PLI, and then extracted network metrics to evaluate the topological characteristics of the networks. It was found that network metrics constructed using the Coh coupling method and CST binarization method showed more significant differences between pre- and post-ECT treatments. However, the network metrics obtained from ICoh/PLI and CST only showed marginally significant differences between pre- and post-ECT patients, which might be affected by the epoch length of EEG signals (Bonita et al., 2014; Fraschini et al., 2016). Though the Coh coupling method was influenced by the artifacts of volume conduction, relevant studies reported that voltage correlation due to volume conduction would be unimportant for electrodes at intervals of 4 cm or more (Doesburg et al., 2005), and a small amount of sparsely distributed electrodes could reduce the effect of volume conduction (Kim et al., 2013). In addition, the network sparsity of CST was significantly higher than that of MST, and the network sparsity of CST was different for every subject (Supplementary Figure 4). The results demonstrated that CST captured differences at low and high thresholds, which might make various network measures more sensitive (Sun et al., 2019). However, MST resulted in a highly sparse network where important edges might be removed, leading to insensitivity to subtle differences in the brain network (Smith et al., 2016). So, for the 19-channel EEG systems, we concluded that combining the coupling method Coh with the binarization technique CST could facilitate the construction of a valid functional brain network.

Based on this combination method, our results found that in the alpha frequency band, network measures (GE, EBC, LE, and meanD) of post-ECT patients with MDD were significantly higher than those of pre-ECT patients with MDD. In addition, increased GE and LE values indicated a general increase in network efficiency. Previous research indicated that compared to normal controls, the shorter characteristic path length and greater GE, as well as lower clustering coefficient and LE, were found in patients with MDD (Li et al., 2015; Li H. et al., 2017). These studies concluded that functional brain networks in subjects with MDD were closer to a randomized network architecture, which might prove that ECT could effectively modulate neuronal network organization and optimize the topological structure of the brain networks in patients with MDD. The results of the evaluation of the network metrics (GE, LE, NBC, EBC, and meanD) in normal controls might reveal that ECT treatment could modulate the abnormal brain pattern of depressive patients tended to normalization, and then improve the efficiency of brain information communication (described in detail in Supplementary Section 6). A recent resting-state fMRI study has indicated that patients with MDD had significantly increased normalized clustering coefficient, normalized path length, and normalized small-worldness after ECT treatment (Sinha et al., 2019). However, an ECT study based on the functional brain network of EEG had reported an opposing conclusion. They found that the brain network of MDD patients deviated from the small-world structure after an ECT session, with a significant increase in the characteristic path length and a significant decrease in the clustering coefficient post-treatment (Deng et al., 2015). However, analysis of only one ECT session might not be able to predict the cumulative neurocognitive changes after multiple ECT treatment sessions, and hence the inconsistent conclusion was also reasonable. Moreover, in Figures 1–5, we can observe that there are individual differences among depressive patients, which may translate into the variability of derived indicators of brain network measures. Hence, a larger sample is required to confirm and generalize our results.

Furthermore, by analyzing the distribution characteristics of hubs, it was found that the hubs mainly existed in the right frontal lobe of patients with MDD before the ECT treatment in the alpha frequency band. These results might reflect the unsuccessful effort of MDD to overcome dysfunction (Rotenberg, 2004). However, after the ECT treatment, the hubs were mainly distributed in the frontal and occipital brain regions of patients with MDD. One relevant study suggested that ECT induced slow-wave oscillations in the frontal brain region (Farzan et al., 2014). Another study found that ECT increased theta-current source density in the ACC and increased connectivity between the anterior frontal cortex and the posterior cingulate cortex regions (Takamiya et al., 2019). Therefore, it is tempting to speculate that ECT might act to rebalance hemispheric activity by regulating the connectivity between frontal and occipital brain regions, thereby alleviating the depressive symptoms.

The results of repeated measures correlation suggested that within the individuals, an increase in the GE, LE, EBC, and meanD values of the alpha frequency band is moderately correlated with a decrease in HAMD-17 scores. From Figure 7, it can be observed that the changes in network metrics and HAMD-17 scores between pre- and post-ECT treatments showed the same trend in most of the patients with MDD, that is, the value of network metrics increased and the depression score decreased. Therefore, we could consider that the increase in network metrics between the pre- and post-ECT treatments correlates with the remission of depressive symptoms. In contrast to repeated measures correlation, the results of Pearson’s correlation showed no association between these changed network metrics and changed HAMD-17 scores. These inconsistent results of the two statistical methods were consistent with the previous studies (Nuninga et al., 2020; Gbyl et al., 2021; Takamiya et al., 2021). In contrast to Pearson correlation analysis, repeated measures correlation estimated the common regression slope, that is, the correlation shared among individuals. Moreover, repeated measures correlation utilized the longitudinal data measured before and after ECT treatment, which greatly improves the statistical accuracy (Bakdash and Marusich, 2017). Therefore, future research studies should try to use repeated measures correlation to reproduce our results.

Several issues need to be addressed further. First, the sample size was relatively small, and there was a lack of comparison with the normal controls under similar experimental conditions and equipment. Hence, larger populations were required to validate and generalize our results. Second, this study used a 19-channel EEG system at the sensor level to investigate functional brain networks. However, to bridge the gap between EEG and fMRI findings of subjects with MDD, high-density EEG (128 and 256 channels) recording combined with source localization technique should be adopted in future studies, as high-density EEG recording could improve topology accuracy (Holmes et al., 2010). Finally, to determine whether the network metrics can act as effective biomarkers to evaluate the efficacy of ECT treatment, future studies may be required to adopt data mining and deep learning algorithms to classify the remission and non-remission patients with MDD after ECT treatment.

## Data Availability Statement

The raw data supporting the conclusions of this article will be made available by the authors, without undue reservation.

## Ethics Statement

The studies involving human participants were reviewed and approved by Jining Medical University. The patients/participants provided their written informed consent to participate in this study.

## Author Contributions

BH, GL, and XL originated the study and revised the manuscript. SS and PY conducted data analysis and drafted the manuscript. SS, PY, and SJ collected the relevant data. SS, HC, and XS conducted pre-processing of data. All authors contributed to and have approved the final manuscript.

## Conflict of Interest

The authors declare that the research was conducted in the absence of any commercial or financial relationships that could be construed as a potential conflict of interest.

## Publisher’s Note

All claims expressed in this article are solely those of the authors and do not necessarily represent those of their affiliated organizations, or those of the publisher, the editors and the reviewers. Any product that may be evaluated in this article, or claim that may be made by its manufacturer, is not guaranteed or endorsed by the publisher.

## Footnotes

1
https://www.nitrc.org/projects/bct/

## References

[B1] Abbott C. C. Lemke N. T. Gopal S. Thoma R. J. Bustillo J. Calhoun V. D. (2013). Electroconvulsive therapy response in major depressive disorder: a pilot functional network connectivity resting state FMRI investigation. *Front. Psychiatr.* 4:10. 10.3389/fpsyt.2013.00010 23459749 PMC3585433

[B2] Akdemir Akar S. Kara S. Agambayev S. Bilgiç V. (2015). Nonlinear analysis of EEGs of patients with major depression during different emotional states. *Comput. Biol. Med.* 67 49–60. 10.1016/j.compbiomed.2015.09.019 26496702

[B3] Alexander-Bloch A. F. Vértes P. E. Stidd R. Lalonde F. Clasen L. Rapoport J. (2013). The anatomical distance of functional connections predicts brain network topology in health and schizophrenia. *Cereb. Cortex.* 23 127–138. 10.1093/cercor/bhr388 22275481 PMC3513955

[B4] Bakdash J. Z. Marusich L. R. (2017). Repeated Measures Correlation. *Front. Psychol.* 8:456. 10.3389/fpsyg.2017.00456 28439244 PMC5383908

[B5] Bohr I. J. Kenny E. Blamire A. O’Brien J. T. Thomas A. J. Richardson J. (2012). Resting-state functional connectivity in late-life depression: higher global connectivity and more long distance connections. *Front. Psychiatr.* 3:116. 10.3389/fpsyt.2012.00116 23316175 PMC3540775

[B6] Bonita J. D. Ambolode L. C. II Rosenberg B. M. Cellucci C. J. Watanabe T. A. Rapp P. E. (2014). Time domain measures of inter-channel EEG correlations: a comparison of linear, nonparametric and nonlinear measures. *Cogn. Neurodyn.* 8 1–15. 10.1007/s11571-013-9267-8 24465281 PMC3890093

[B7] Bouckaert F. De Winter F. L. Emsell L. Dols A. Rhebergen D. Wampers M. (2016). Grey matter volume increase following electroconvulsive therapy in patients with late life depression: a longitudinal MRI study. *J. Psychiatr. Neurosci.* 41 105–114. 10.1503/jpn.140322 26395813 PMC4764479

[B8] Brunoni A. R. Moffa A. H. Fregni F. Palm U. Padberg F. Blumberger D. M. (2016). Transcranial direct current stimulation for acute major depressive episodes: meta-analysis of individual patient data. *Br. J. Psychiatr.* 208 522–531. 10.1192/bjp.bp.115.164715 27056623 PMC4887722

[B9] Bullmore E. Sporns O. (2009). Complex brain networks: graph theoretical analysis of structural and functional systems. *Nat. Rev. Neurosci.* 10 186–198. 10.1038/nrn2575 19190637

[B10] Croft R. J. Barry R. J. (2000). EOG correction: which regression should we use? *Psychophysiology* 37 123–125. 10705774

[B11] Deng Z. D. McClinctock S. M. Lisanby S. H. (2015). Brain network properties in depressed patients receiving seizure therapy: a graph theoretical analysis of peri-treatment resting EEG. *Annu. Int. Conf. IEEE Eng. Med. Biol. Soc.* 2015 2203–2206. 10.1109/EMBC.2015.7318828 26736728

[B12] Doesburg S. M. Kitajo K. Ward L. M. (2005). Increased gamma-band synchrony precedes switching of conscious perceptual objects in binocular rivalry. *Neuroreport* 16 1139–1142. 10.1097/00001756-200508010-00001 16012336

[B13] Farzan F. Atluri S. Mei Y. Moreno S. Levinson A. J. Blumberger D. M. (2017). Brain temporal complexity in explaining the therapeutic and cognitive effects of seizure therapy. *Brain* 140 1011–1025. 10.1093/brain/awx030 28335039

[B14] Farzan F. Boutros N. N. Blumberger D. M. Daskalakis Z. J. (2014). What does the electroencephalogram tell us about the mechanisms of action of ECT in major depressive disorders? *J. Ect.* 30 98–106. 10.1097/YCT.0000000000000144 24810774

[B15] Fingelkurts A. A. Fingelkurts A. A. Kähkönen S. (2005). Functional connectivity in the brain–is it an elusive concept? *Neurosci. Biobehav. Rev.* 28 827–836. 10.1016/j.neubiorev.2004.10.009 15642624

[B16] Fraschini M. Demuru M. Crobe A. Marrosu F. Stam C. J. Hillebrand A. (2016). The effect of epoch length on estimated EEG functional connectivity and brain network organisation. *J. Neural. Eng.* 13:036015. 10.1088/1741-2560/13/3/036015 27137952

[B17] Freeman L. C. (1978). Centrality in social networks conceptual clarification. *Soc. Netw.* 1 215–239.

[B18] Gbyl K. Rostrup E. Raghava J. M. Andersen C. Rosenberg R. Larsson H. B. W. (2021). Volume of hippocampal subregions and clinical improvement following electroconvulsive therapy in patients with depression. *Prog. Neuropsychopharmacol. Biol. Psychiatry.* 104:110048. 10.1016/j.pnpbp.2020.110048 32730916

[B19] Gu L. Xie J. Long J. Chen Q. Chen Q. Pan R. (2013). Epidemiology of major depressive disorder in mainland china: a systematic review. *PLoS One* 8:e65356. 10.1371/journal.pone.0065356 23785419 PMC3681935

[B20] Hamilton M. (1967). Development of a rating scale for primary depressive illness. *Br. J. Soc. Clin. Psychol.* 6 278–296. 6080235 10.1111/j.2044-8260.1967.tb00530.x

[B21] He Y. Evans A. (2010). Graph theoretical modeling of brain connectivity. *Curr. Opin. Neurol.* 23 341–350. 10.1097/WCO.0b013e32833aa567 20581686

[B22] Hill A. T. Zomorrodi R. Hadas I. Farzan F. Voineskos D. Throop A. (2021). Resting-state electroencephalographic functional network alterations in major depressive disorder following magnetic seizure therapy. *Prog. Neuropsychopharmacol. Biol. Psychiatr.* 108:110082. 10.1016/j.pnpbp.2020.110082 32853716

[B23] Holmes M. D. Tucker D. M. Quiring J. M. Hakimian S. Miller J. W. Ojemann J. G. (2010). Comparing noninvasive dense array and intracranial electroencephalography for localization of seizures. *Neurosurgery* 66 354–362. 10.1227/01.NEU.0000363721.06177.07 20087136

[B24] Hosseini S. M. Hoeft F. Kesler S. R. (2012). GAT: a graph-theoretical analysis toolbox for analyzing between-group differences in large-scale structural and functional brain networks. *PLoS One* 7:e40709. 10.1371/journal.pone.0040709 22808240 PMC3396592

[B25] Keller M. B. (2003). Past, present, and future directions for defining optimal treatment outcome in depression: remission and beyond. *Jama* 289 3152–3160. 10.1001/jama.289.23.3152 12813121

[B26] Kellner C. H. McClintock S. M. McCall W. V. Petrides G. Knapp R. G. Weiner R. D. (2014). Brief pulse and ultrabrief pulse right unilateral electroconvulsive therapy (ECT) for major depression: efficacy, effectiveness, and cognitive effects. *J. Clin. Psychiatr* 75:777. 10.4088/JCP.14lr08997 25093475 PMC7059912

[B27] Kim D. J. Bolbecker A. R. Howell J. Rass O. Sporns O. Hetrick W. P. (2013). Disturbed resting state EEG synchronization in bipolar disorder: a graph-theoretic analysis. *Neuroimage Clin.* 2 414–423. 10.1016/j.nicl.2013.03.007 24179795 PMC3777715

[B28] Latora V. Marchiori M. (2001). Efficient behavior of small-world networks. *Phys. Rev. Lett.* 87:198701. 10.1103/PhysRevLett.87.198701 11690461

[B29] Leuchter A. F. Cook I. A. Hunter A. M. Cai C. Horvath S. (2012). Resting-state quantitative electroencephalography reveals increased neurophysiologic connectivity in depression. *PLoS One.* 7:e32508. 10.1371/journal.pone.0032508 22384265 PMC3286480

[B30] Li H. Zhou H. Yang Y. Wang H. Zhong N. (2017). More randomized and resilient in the topological properties of functional brain networks in patients with major depressive disorder. *J. Clin. Neurosci.* 44 274–278. 10.1016/j.jocn.2017.06.037 28694044

[B31] Li X. Hu B. Sun S. Cai H. (2016). EEG-based mild depressive detection using feature selection methods and classifiers. *Comput. Methods Progr. Biomed.* 136 151–161. 10.1016/j.cmpb.2016.08.010 27686712

[B32] Li X. Jing Z. Hu B. Jing Z. Zhong N. Li M. (2017). A Resting-State Brain Functional Network Study in MDD Based on Minimum Spanning Tree Analysis and the Hierarchical Clustering. *Complexity* 2017 1–11.

[B33] Li Y. Cao D. Wei L. Tang Y. Wang J. (2015). Abnormal functional connectivity of EEG gamma band in patients with depression during emotional face processing. *Clin. Neurophysiol.* 126 2078–2089. 10.1016/j.clinph.2014.12.026 25766267

[B34] Lisanby S. H. (2007). Electroconvulsive therapy for depression. *N. Engl. J. Med.* 357 1939–1945. 17989386 10.1056/NEJMct075234

[B35] Mayberg H. S. Lozano A. M. Voon V. McNeely H. E. Seminowicz D. Hamani C. (2005). Deep brain stimulation for treatment-resistant depression. *Neuron* 45 651–660. 15748841 10.1016/j.neuron.2005.02.014

[B36] McCormick L. M. Yamada T. Yeh M. Brumm M. C. Thatcher R. W. (2009). Antipsychotic effect of electroconvulsive therapy is related to normalization of subgenual cingulate theta activity in psychotic depression. *J. Psychiatr. Res.* 43 553–560. 10.1016/j.jpsychires.2008.08.004 18851858

[B37] Nuninga J. O. Mandl R. C. W. Boks M. P. Bakker S. Somers M. Heringa S. M. (2020). Volume increase in the dentate gyrus after electroconvulsive therapy in depressed patients as measured with 7T. *Mol. Psychiatr.* 25 1559–1568. 10.1038/s41380-019-0392-6 30867562

[B38] Okazaki R. Takahashi T. Ueno K. Takahashi K. Higashima M. Wada Y. (2013). Effects of electroconvulsive therapy on neural complexity in patients with depression: report of three cases. *J. Affect. Disord.* 150 389–392. 10.1016/j.jad.2013.04.029 23701750

[B39] Pedroni A. Bahreini A. Langer N. (2019). Automagic: standardized preprocessing of big EEG data. *Neuroimage* 200 460–473. 10.1016/j.neuroimage.2019.06.046 31233907

[B40] Perrin J. S. Merz S. Bennett D. M. Currie J. Steele D. J. Reid I. C. (2012). Electroconvulsive therapy reduces frontal cortical connectivity in severe depressive disorder. *Proc. Natl. Acad. Sci. U S A.* 109 5464–5468. 10.1073/pnas.1117206109 22431642 PMC3325678

[B41] Reischies F. M. Neuhaus A. H. Hansen M. L. Mientus S. Mulert C. Gallinat J. (2005). Electrophysiological and neuropsychological analysis of a delirious state: the role of the anterior cingulate gyrus. *Psychiatr. Res.* 138 171–181. 10.1016/j.pscychresns.2004.06.008 15766639

[B42] Ritchey M. Dolcos F. Eddington K. M. Strauman T. J. Cabeza R. (2011). Neural correlates of emotional processing in depression: changes with cognitive behavioral therapy and predictors of treatment response. *J. Psychiatr. Res.* 45 577–587. 10.1016/j.jpsychires.2010.09.007 20934190 PMC3042483

[B43] Rotenberg V. S. (2004). The peculiarity of the right-hemisphere function in depression: solving the paradoxes. *Prog. Neuropsychopharmacol. Biol. Psychiatry.* 28 1–13. 10.1016/S0278-5846(03)00163-5 14687851

[B44] Rubinov M. Sporns O. (2010). Complex network measures of brain connectivity: uses and interpretations. *Neuroimage* 52 1059–1069. 10.1016/j.neuroimage.2009.10.003 19819337

[B45] Sakkalis V. (2011). Review of advanced techniques for the estimation of brain connectivity measured with EEG/MEG. *Comput. Biol. Med.* 41 1110–1117. 10.1016/j.compbiomed.2011.06.020 21794851

[B46] Sartorius N. (2001). The economic and social burden of depression. *J. Clin. Psychiatr.* 62(Suppl. 15), 8–11. 11444765

[B47] Sheline Y. I. Price J. L. Yan Z. Mintun M. A. (2010). Resting-state functional MRI in depression unmasks increased connectivity between networks via the dorsal nexus. *Proc. Natl. Acad. Sci. U S A.* 107 11020–11025. 10.1073/pnas.1000446107 20534464 PMC2890754

[B48] Siegle G. J. Carter C. S. Thase M. E. (2006). Use of FMRI to predict recovery from unipolar depression with cognitive behavior therapy. *Am. J. Psychiatr.* 163 735–738. 10.1176/appi.ajp.163.4.735 16585452

[B49] Sinha P. Reddy R. V. Srivastava P. Mehta U. M. Bharath R. D. (2019). Network neurobiology of electroconvulsive therapy in patients with depression. *Psychiatr. Res. Neuroimaging* 287 31–40. 10.1016/j.pscychresns.2019.03.008 30952030

[B50] Smith K. Abasolo D. Escudero J. (2016). A comparison of the cluster-span threshold and the union of shortest paths as objective thresholds of EEG functional connectivity networks from Beta activity in Alzheimer’s disease. *Ann. Int. Conf. IEEE Eng. Med. Biol. Soc.* 2016 2826–2829. 10.1109/EMBC.2016.7591318 28268906

[B51] Smith K. Abásolo D. Escudero J. (2017). Accounting for the complex hierarchical topology of EEG phase-based functional connectivity in network binarisation. *PLoS One* 12:e0186164. 10.1371/journal.pone.0186164 29053724 PMC5650149

[B52] Smith K. Azami H. Parra M. A. Starr J. M. Escudero J. (2015). Cluster-span threshold: an unbiased threshold for binarising weighted complete networks in functional connectivity analysis. *Annu. Int. Conf. IEEE Eng. Med. Biol. Soc.* 2015 2840–2843. 10.1109/EMBC.2015.7318983 26736883

[B53] Spronk D. Arns M. Barnett K. J. Cooper N. J. Gordon E. (2011). An investigation of EEG, genetic and cognitive markers of treatment response to antidepressant medication in patients with major depressive disorder: a pilot study. *J. Affect. Disord.* 128 41–48. 10.1016/j.jad.2010.06.021 20619899

[B54] Sun S. Li X. Zhu J. Wang Y. La R. Zhang X. (2019). Graph Theory Analysis of Functional Connectivity in Major Depression Disorder With High-Density Resting State EEG Data. *IEEE Trans. Neural Syst. Rehabil. Eng.* 27 429–439. 10.1109/TNSRE.2019.2894423 30676968

[B55] Takamiya A. Hirano J. Yamagata B. Takei S. Kishimoto T. Mimura M. (2019). Electroconvulsive Therapy Modulates Resting-State EEG Oscillatory Pattern and Phase Synchronization in Nodes of the Default Mode Network in Patients With Depressive Disorder. *Front. Hum. Neurosci.* 13:1. 10.3389/fnhum.2019.00001 30774588 PMC6367251

[B56] Takamiya A. Kishimoto T. Hirano J. Kikuchi T. Yamagata B. Mimura M. (2021). Association of electroconvulsive therapy-induced structural plasticity with clinical remission. *Prog. Neuropsychopharmacol. Biol. Psychiatr.* 110:110286. 10.1016/j.pnpbp.2021.110286 33621611

[B57] van Diessen E. Numan T. van Dellen E. van der Kooi A. W. Boersma M. Hofman D. (2015). Opportunities and methodological challenges in EEG and MEG resting state functional brain network research. *Clin. Neurophysiol.* 126 1468–1481. 10.1016/j.clinph.2014.11.018 25511636

[B58] Wei Q. Bai T. Brown E. C. Xie W. Chen Y. Ji G. (2020). Thalamocortical connectivity in electroconvulsive therapy for major depressive disorder. *J. Affect. Disord.* 264 163–171. 10.1016/j.jad.2019.11.120 32056746

[B59] Wei Q. Bai T. Chen Y. Ji G. Hu X. Xie (2018). The Changes of Functional Connectivity Strength in Electroconvulsive Therapy for Depression: a Longitudinal Study. *Front. Neurosci.* 12:661. 10.3389/fnins.2018.00661 30319341 PMC6167462

[B60] Winkler I. Haufe S. Tangermann M. (2011). Automatic classification of artifactual ICA-components for artifact removal in EEG signals. *Behav. Brain Funct.* 7:30. 10.1186/1744-9081-7-30 21810266 PMC3175453

[B61] Yrondi A. Sporer M. Péran P. Schmitt L. Arbus C. Sauvaget A. (2018). Electroconvulsive therapy, depression, the immune system and inflammation: a systematic review. *Brain Stimul.* 11 29–51. 10.1016/j.brs.2017.10.013 29111078

