# Supplementary material for: Electroconvulsive Therapy-Induced Changes in Functional Brain Network of Major Depressive Disorder Patients: A Longitudinal Resting-State Electroencephalography Study
Source: Front Hum Neurosci. 2022 May 18;16:852657. doi: 10.3389/fnhum.2022.852657 (PMC9158117; doi:10.3389/fnhum.2022.852657)
Supplement: Supplementary file 1 [file Data_Sheet_1.docx]

Supplementary Material

Table of Contents

[1 Coupling methods 1](#_Toc99839269)

[1.1 Coherence (Coh) and Imaginary part of coherency (ICoh) 1](#_Toc99839270)

[1.2 Phase lag index (PLI) 2](#_Toc99839271)

[2 Binarization methods 2](#_Toc99839272)

[2.1 Cluster-Span Threshold (CST) 2](#_Toc99839273)

[2.2 Minimum Spanning Tree (MST) 3](#_Toc99839274)

[3 Network metrics 3](#_Toc99839275)

[3.1 Global Efficiency (GE) 3](#_Toc99839276)

[3.2 Edge Betweenness Centrality (EBC) 3](#_Toc99839277)

[3.3 Node Betweenness Centrality (NBC) 3](#_Toc99839278)

[3.4 Local Efficiency (LE) 4](#_Toc99839279)

[3.5 Mean Degree (meanD) 4](#_Toc99839280)

[4 ECT-induced changes in network metrics 4](#_Toc99839281)

[5 Correlation between changes in network metrics and clinical response 8](#_Toc99839282)

[6 Control evaluation of network metrics 9](#_Toc99839283)

[References 11](#_Toc99839284)

# Coupling methods

## 1.1 Coherence (Coh) and Imaginary part of coherency (ICoh)

Coherency is a measure of linear relationship between two EEG-channels signals at a specific frequency [1]. Coh and ICoh can obtained from complex coherency. Given the two electrodes i and j at frequency f, the complex coherency can be obtained as

| $C_{xy}\left( f \right)= \frac{P_{xy}\left( f \right)}{\sqrt{P_{xx}\left( f \right)P_{yy}\left( f \right)}}=Real\left\{ Cxy\left( f \right) \right\}+Imag\{Cxy\left( f \right)\}$ | (1) |
| --- | --- |

Where $P_{xy}\left( f \right)$ is the cross-power spectral density (PSD) estimate of the electrode signals x and y at the frequency of f, and $P_{xx}\left( f \right)$ or $P_{yy}\left( f \right)$ is the PSD estimate of electrode signal x or y at the frequency of f. $Real\left\{ C_{xy}\left( f \right) \right\}$ and $Imag\{C_{xy}\left( f \right)\}$ respectively denotes real part and imaginary part of $C_{xy}\left( f \right)$.

Hence, Coh is defined as the absolute value of the real part of coherency:

|  | $\mathrm{Coh}_{xy}(f)={\vert C}_{xy}\left( f \right)\vert$ | (2) |
| --- | --- | --- |

ICoh is defined as the imaginary part of coherency:

|  | $\mathrm{ICoh}_{xy}(f)={imag(C}_{xy}\left( f \right))$ | (3) |
| --- | --- | --- |

Fisher’s Z transformation is applied to Coh and ICoh to ensure an approximate normal distribution [2].

In this study, the dimension of Coh and ICoh is 16*16, 16 is the number of electrode channels, the row x and column y represents the connectivity strength of channels x and y which is estimated by averaging connectivity strength within the frequency band raged from f1 to f2. The frequency bands include delta band (1 - 4Hz), theta band (4 - 8Hz), alpha band (8 - 13Hz) and beta band (13 - 30Hz). The value of Coh/ICoh is between [0, 1], where 1 indicates maximum linear interdependence and 0 indicates no linear interdependence.

## 1.2 Phase lag index (PLI)

PLI [3] is used to estimate the asymmetry of the phase differences distribution between two-channel EEG signals, PLI is defined as:

|  | $\mathrm{PLI}_{xy}\left( f \right)=\vert<sign(\emptyset_{x}\left( f \right)-\emptyset_{y}\left( f \right))>\vert$ | (4) |
| --- | --- | --- |

Where <> represents expectation value, $\emptyset_{x}\left( f \right)-\emptyset_{y}(f)$ represents the phase synchronization between the signals in channel x and y at the frequency of f. It’s essential to know the instantaneous phase of the two signals involved, which can be completed using the analytical signal based on the Hilbert transform [4], so as to compute the phase synchronization. The frequency bands of interest include delta band (1 - 4Hz), theta band (4 - 8Hz), alpha band (8 - 13Hz) and beta band (13 - 30Hz). The value of $\mathrm{PLI}_{xy}\left( f \right)$ is between [0, 1], where 1 indicates perfect phase synchronization, 0 indicates no coupling.

# Binarization methods

## 2.1 Cluster-Span Threshold (CST)

CST [5, 6] selects the threshold by adjusting the ratio of closed to open triples to reach balance. We calculated the binarized networks within a wide range of density (15% to 85% in step of 1%) and chose the one for which the number of strongest links present obtained the global clustering co-efficiency $C_{Glob\left( density \right)}$ nearest to 0.5, when

|  | $C_{Glob(density)}= \sum_{i} \frac{(M^{3})_{ii}}{\sum_{j} T_{ij}}\approx0.5$ | (1) |
| --- | --- | --- |

Where

|  | $T=M^{2}-Diag(M^{2})$ | (2) |
| --- | --- | --- |

Where M is a weighted adjacency matrix, M_ij_ represents the entry of i_th_ row and j_th_ column of M. T is the matrix of triples between nodes, T_ij_ represents the number of triples beginning at node i and ending at node j. $Diag(M^{2})$ is the matrix of M^2^ that preserves the diagonal values, while sets the rest of values to 0. So T is the matrix of $M^{2}$ with the diagonal entries set to 0.

Because the graphs are the discrete, the ratio of closed to open triples is unlikely to obtain 0.5. Therefore, we chose the CST when satisfying Eq. (7) that could obtain the minimum absolute value through $C_{Glob\left( density \right)}$ minus the clustering coefficient value which obtains an equilibrium between closed and open triples 0.5 , where

|  | $Z=argmin\left( abs\left( C_{Glob\left( density \right)}-0.5 \right) \right)$ | (3) |
| --- | --- | --- |

## 2.2 Minimum Spanning Tree (MST)

MST is an acyclic sub-network that contains all nodes while minimizing the link weights. Two algorithms have been used to constructed the MST based on the weighted networks [7, 8]. In our study Kruskal's algorithm was adopted to construct the MST of maximum the sum of the link weights, which means we are interested in the strongest connections in the network. To construct the MST of the weighted graph with N nodes, we first rank the weights of all links in the graph from highest to lowest and we start with N disconnected nodes. The largest weight link connects two nodes is chose to form the first maximum tree. Then the following largest weight link will be added until all N nodes are connected in a no loop sub-network, in the final this sub-network will have N-1 links. In the process, when adding a new link will form a loop, this link is discarded, the procedure continues by selecting the next sequential weight link. After construction of the MST, all link weights in this sub-network are set a value of one.

# Network metrics

## 3.1 Global Efficiency (GE)

GE is the average inverse value of shortest path lengths between all nodes in the network.

|  | $GE= \frac{1}{N}\sum_{i=1}^{N} E_{i}= \frac{1}{N}\sum_{i=1}^{N} \frac{\sum_{j\in N,j\neq i} d_{ij}^{-1}}{N-1}$ | (1) |
| --- | --- | --- |

Where N is the number of nodes. $E_{i}$ is the efficiency of node i. $d_{ij}$ is the shortest path length between node i and node j.

## 3.2 Edge Betweenness Centrality (EBC)

EBC is the fraction of all shortest paths in the network that pass through a given edge.

|  | $\mathrm{EBC}_{ij}= \sum_{\begin{aligned} h,k\in N, \\ h\neq k \end{aligned}} \frac{\rho_{hk}(e_{ij})}{\rho_{hk}}$ | (2) |
| --- | --- | --- |

Where $e_{ij}$ is the connection edge between nodes i and j, $\rho_{hk}$ is the number of shortest paths between nodes h and k, $\rho_{hk}(e_{ij})$ is the number of shortest paths between nodes h and k that pass through edge $e_{ij}$.

## 3.3 Node Betweenness Centrality (NBC)

NBC is the number of shortest paths in the network that make use of a given node.

|  | $\mathrm{NBC}_{i}= \sum_{\begin{aligned} h,j\in N, \\ h\neq j\neq i \end{aligned}} \frac{\rho_{hj}(i)}{\rho_{hj}}$ | (3) |
| --- | --- | --- |

Where $\rho_{hj}$ is the number of shortest paths between nodes h and j, $\rho_{hj}(i)$ is the number of shortest paths between nodes h and j that make use of node i.

## 3.4 Local Efficiency (LE)

LE of node i is calculated as

|  | $\mathrm{LE}_{i}= \frac{1}{d_{i}(d_{i}-1)}\sum_{j,k=G_{i}} \frac{1}{d_{jk}}$ | (4) |
| --- | --- | --- |

Where $d_{i}$ is the degree of node i, $G_{i}$ is the subgraph of the neighbor nodes of node i excluding node i. LE is average of local efficiency of all nodes.

|  | $LE = \frac{1}{N}\sum_{i=1}^{N} {LE}_{i}$ | (5) |
| --- | --- | --- |

## 3.5 Mean Degree (meanD)

The degree of a node $d_{i}$is the number of links connected to it.

|  | $d_{i}= \sum_{i=1}^{N} a_{ij}$ | (6) |
| --- | --- | --- |

$a_{ij}$ is the connection status between node i and node j, $a_{ij}=1$, if the edge (i,j) exists; otherwise $a_{ij}=0$.

The meanD is the average of all nodes degree.

|  | $meanD= \frac{1}{N}\sum_{i=1}^{N} d_{i}$ | (7) |
| --- | --- | --- |

# ECT-induced changes in network metrics

When the connectivity values were estimated by Coh, ICoh or PLI, there were no differences in different network metrics pre- and post-ECT MDD patients in four frequency bands, when MST was used for binarization (see Supplementary Figure 1 to 3). What should be noted was that LE calculated from network constructed by MST was zero, due to MST was a no-looping graph. When a node is removed from its neighbouring set, they will be disconnected with zero LE. And meanD calculated from network constructed by MST was 1.875 ((16 - 1) * 2 / 16).

**Supplementary Figure 1.** Network metrics of pre- and post-ECT MDD patients obtained by coupling method Coherence (Coh) and binarization approach Minimum Spanning Tree (MST) in delta, theta, alpha and beta frequency bands. Network metrics include global efficiency (GE), edge betweenness centrality (EBC), node betweenness centrality (NBC), local efficiency (LE) and mean degree (meanD). The bar chart represents the mean value of network metrics in pre- and post-ECT MDD patients. The ***** indicates a significant difference (p < 0.05), the ***’** represent marginally significant difference (0.05 < p < 0.1), Pseudo paired t-test was conducted based on permutation test n = 10000, p = 0.05.

**Supplementary Figure 2.** Network metrics of pre- and post-ECT MDD patients obtained by coupling method imaginary part of coherence (ICoh) and binarization approach Minimum Spanning Tree (MST) in delta, theta, alpha and beta frequency bands. Other descriptions are as in **Supplementary Figure 1**.

**Supplementary Figure 3.** Network metrics of pre- and post-ECT MDD patients obtained by coupling method phase lag index (PLI) and binarization approach Minimum Spanning Tree (MST) in delta, theta, alpha and beta frequency bands. Other descriptions are as in **Supplementary Figure 1**.

The comparison of network sparsity between CST and MST was shown in Supplementary Figure 4. It could be found that the network sparsity of CST was significantly higher than that of MST. What should be noted was that MST was a no loop sub-network with N-1 links. The network sparsity of MST was 12.5% (15 / (16 * (16 - 1) / 2)). And the network sparsity of CST for every subject is different. The results confirmed that CST chose the threshold by adjusting the ratio of closed to open triples to reach balance, rather than fixing connection density at an arbitrary value. This method could ensure a trade-off of sparsity and density of information [9]. CST captured the differences found at both high and low threshold levels, which might make different network metrics become more sensitive [9]. However, MST led to highly sparse networks, thus some important connections information might be absent, in turn resulting in insensitive to more subtle differences in cognitive function [10]. Therefore, compared with MST, CST was a better binarization method.

**Supplementary Figure 4.** Comparison of network sparsity between CST and MST binarization methods with a combination of three coupling methods of pre- and post-ECT MDD patients in four frequency bands.

# Correlation between changes in network metrics and clinical response

Pearson correlation was used to estimate the relationship between changes in network metrics (GE, LE, EBC and meanD) and clinical response. It was found that changed network metrics had no significant correlation with changed HAMD-17 scores (see Supplementary Figure 5).


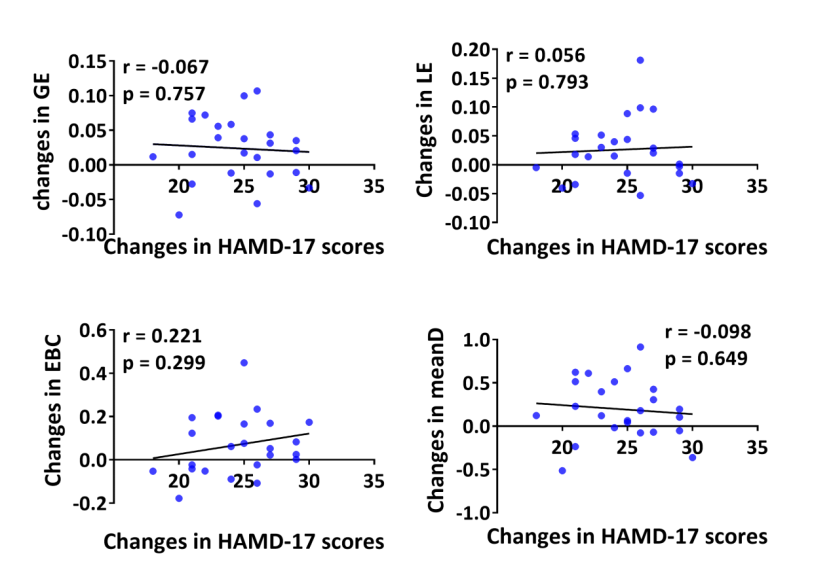


**Supplementary Figure 5.** Relationship between the network metrics and the clinical response in individual MDD patients

# Control evaluation of network metrics

In order to evaluate the network metrics (GE, LE, NBC, EBC, meanD) in normal controls (NC), we used MODMA (Multi-modal Open Dataset for Mental-disorder Analysis) dataset including patients with major depressive disorder (MDD) and NC. The NC group matched the age and gender of MDD patients in this study. The demographic characteristics of MDD patients and NC are shown in Supplementary Table 1.

For each normal subject, eye-closed resting state EEG signals were continuously recorded for approximately 5 min using a 128 channel HydroCel Geodesic Sensor Net (HCGSN) with a Cz reference. The sampling frequency was 250 Hz with Net Station acquisition software and Electrical Geodesics amplifiers. Electrode impedance was kept below 50 kΩ. To ensure the same number of electrode channels, we selected the same 16 electrodes (Fp1/2, F3/4, C3/4, P3/4, O1/2, F7/8, T3/4, T5/6, Fz, Cz, Pz) from 128 electrodes (electrode number: E9, E22, E24, E33, E36, E45, E52, E58, E70, E83, E92, E96, E104, E108, E122, E124). And preprocessing method was same as what we used in the manuscript. The coupling method Coh and binarization method CST were also used to constructed functional brain networks, because the effectiveness of this method had been proved in the manuscript.

Due to there were significant differences between pre- and post-ECT MDD patients in network metrics (GE, LE, NBC, EBC, meanD) of alpha band, we conducted statistical analysis (non-parametric permutation test) of these network metrics between pre- and post-ECT MDD patients and NC in the alpha band. From Supplementary Figure 6, we found that relative to the NC group, the pre-ECT MDD group had significantly lower GE (p < 0.05), NBC (p < 0.05), EBC (p < 0.05) and marginally significant lower meanD (0.05 < p < 0.1). The results were consistent with previous findings[9, 11, 12]. However, after ECT treatment, although these network metrics of post-ECT patients were still lower that of NC, there were no significant differences in GE and meanD between post-ECT MDD patients and NC. And after ECT treatment, statistical result of network metric NBC between MDD and NC groups was transformed from having significant differences (p < 0.05) to having marginally significant differences (0.05 < p < 0.1). In addition, we found that statistical results of some network attributes (LE and EBC) did not change significantly between MDD and NC groups before and after ECT treatment, but we found that compared to pre-ECT MDD patients, the network metrics of post-ECT MDD patients were improved. So we could speculate that ECT treatment could modulate the abnormal brain pattern of depressive patients tended to normalization, and then improve the efficiency of brain information communication.

**Supplementary Table 1.** The demographic variables of MDD patients and matched NC

|  | **MDD** | **NC** | **P value** |
| --- | --- | --- | --- |
| **Age** | 33.54 ± 13.75 | 34.08 ± 9.96 | 0.88 |
| **Gender** | 15M/9F | 15M/9F | 1 |


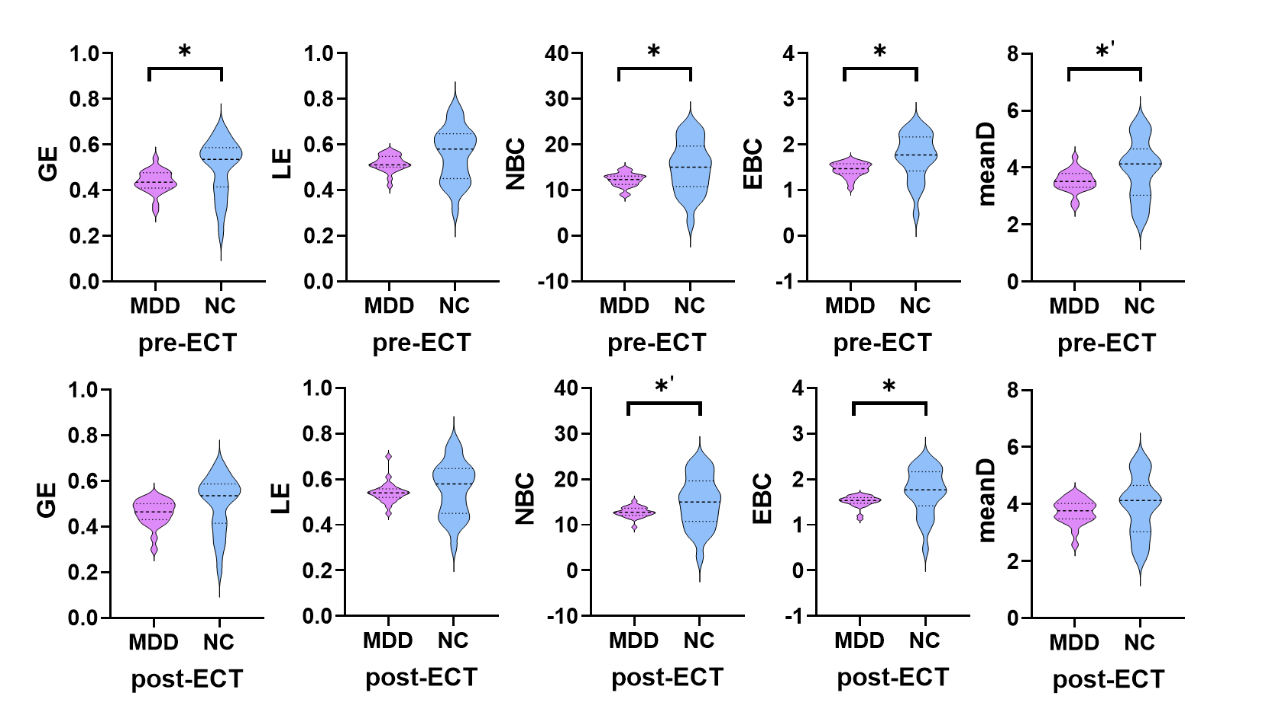


**Supplementary Figure 6.** Statistical results of network metrics between pre- and post-ECT MDD patients and NC in the alpha band. The * indicates a significant difference (p < 0.05), the *’ represent marginally significant difference (0.05 < p < 0.1), non-parametric permutation test was conducted (n = 10000, p = 0.05).

# References

1. Nunez, P.L., et al., *EEG coherency : I: statistics, reference electrode, volume conduction, Laplacians, cortical imaging, and interpretation at multiple scales.* Electroencephalogr Clin Neurophysiol, 1997. **103**(5): p. 499-515.

2. Nolte, G., et al., *Identifying true brain interaction from EEG data using the imaginary part of coherency.* Clinical Neurophysiology, 2004. **115**(10): p. 2292-2307.

3. Stam, C.J., G. Nolte, and A. Daffertshofer, *Phase lag index: assessment of functional connectivity from multi channel EEG and MEG with diminished bias from common sources.* Human Brain Mapping, 2007. **28**(11): p. 1178.

4. Bruns, A., *Fourier-, Hilbert- and wavelet-based signal analysis: are they really different approaches?* Journal of Neuroscience Methods, 2004. **137**(2): p. 321.

5. Smith, K., D. Abásolo, and J.J.P.o. Escudero, *Accounting for the complex hierarchical topology of EEG phase-based functional connectivity in network binarisation.* PloS one, 2017. **12**(10): p. e0186164.

6. Smith, K., et al. *Cluster-span threshold: An unbiased threshold for binarising weighted complete networks in functional connectivity analysis*. in *2015 37th Annual International Conference of the IEEE Engineering in Medicine and Biology Society (EMBC)*. 2015. IEEE.

7. Kruskal, J.B., *On the Shortest Spanning Subtree of a Graph and the Traveling Salesman Problem.* Proceedings of the American Mathematical Society, 1956. **7**(1): p. 48-50.

8. Prim, R.C., *Shortest connection networks and some generalizations.* Bell Labs Technical Journal, 2013. **36**(6): p. 1389-1401.

9. Sun, S., et al., Graph Theory Analysis of Functional Connectivity in Major Depression Disorder With High-Density Resting State EEG Data. IEEE Transactions on Neural Systems Rehabilitation Engineering, 2019. 27(3): p. 429-439.

10. Smith, K., D. Abasolo, and J. Escudero. *A comparison of the cluster-span threshold and the union of shortest paths as objective thresholds of EEG functional connectivity networks from Beta activity in Alzheimer's disease*. in *2016 38th Annual International Conference of the IEEE Engineering in Medicine and Biology Society (EMBC)*. 2016.

11. Zhang, M., et al., *Randomized EEG functional brain networks in major depressive disorders with greater resilience and lower rich-club coefficient.* Clinical Neurophysiology, 2018. **129**(4): p. 743-758.

12. Li, Y., et al., *Abnormal functional connectivity of EEG gamma band in patients with depression during emotional face processing.* Clinical Neurophysiology, 2015. **126**(11): p. 2078-89.
